# Screening of spider mites (Acari: Tetranychidae) for reproductive endosymbionts reveals links between co-infection and evolutionary history

**DOI:** 10.1038/srep27900

**Published:** 2016-06-13

**Authors:** Yan-Kai Zhang, Ya-Ting Chen, Kun Yang, Ge-Xia Qiao, Xiao-Yue Hong

**Affiliations:** 1 Institute of Zoology, Chinese Academy of Sciences, Beijing, 100101, China; 2 Department of Entomology, Nanjing Agricultural University, Nanjing, Jiangsu 210095, China

## Abstract

Reproductive endosymbionts have been shown to have wide-ranging effects on many aspects of their hosts’ biology. A first step to understanding how these endosymbionts interact with their hosts is to determine their incidences. Here, we screened for four reproductive endosymbionts (*Wolbachia*, *Cardinium*, *Spiroplasma* and *Rickettsia*) in 28 populations of spider mites (Acari: Tetranychidae) representing 12 species. Each of the four endosymbionts were identified in at least some of the tested specimens, and their infection patterns showed variations at the species-level and population-level, suggesting their distributions can be correlated with both the phylogeny and ecology of the hosts. Co-infections of unrelated bacteria, especially double infections of *Wolbachia* and *Cardinium* within the same individuals were common. *Spiroplasma* and *Rickettsia* infections were specific to particular host species, respectively. Further, the evolutionary histories of these endosymbionts were inferred by comparing the phylogenies of them and their hosts. These findings can help to clarify the interactions between endosymbionts and arthropods.

Symbiotic bacteria are ubiquitous and have profound impacts on their host’s biology^1^,^2^,^3^,^4^. The interest in the field has come largely from the discovery of *Wolbachia*, a bacterium that manipulates hosts’ reproduction through cytoplasmic incompatibility (CI), parthenogenesis, male-killing, feminization^5^ and oogenesis^6^. Within the last decade, a second symbiotic bacterium, *Cardinium*, has been found to have reproductive effects, including cytoplasmic incompatibility^7^,^8^, parthenogenesis^9^ and feminization^10^. Other reproductive endosymbionts have been recorded in the genera *Spiroplasma* and *Rickettsia*. *Spiroplasma* induces male-killing in *Drosophila*^11^, butterflies^12^, ladybird beetles^13^ and planthoppers^14^. *Rickettsia* has been shown to manipulate the reproductive biology of wasps^15^ and beetles^16^,^17^. In addition, many of them also may provide direct fitness benefits to infected individuals, such as protection from pathogens^18^ or increasing fecundity^19^ under certain circumstances. These endosymbionts should thus be recognized as important components of arthropod biology.

*Wolbachia* is widespread in arthropods^20^ and its distribution is related to host ecology and host biology^21^,^22^. Some lineages of *Wolbachia* (termed supergroups A and B) have spread ubiquitously, while others (e.g., supergroups C and D) are taxon-specific. *Cardinium* infections are rarer than *Wolbachia*, and are restricted to Hymenoptera, Hemiptera, Diptera and Acari^23^,^24^,^25^. Double infections of *Wolbachia* and *Cardinium* within the same host species have been found^8^,^26^,^27^. The distributions of *Spiroplasma* and *Rickettsia* and other reproductive bacteria have been widely investigated in some arthropods^28^,^29^,^30^.

In view of the wide distribution of endosymbionts in arthropods and their potential influences on hosts, much remains to be learned about host-bacteria interactions. Spider mites (Acari: Tetranychidae) represent a distinctive evolutionary group that is comprised of about 1200 species, including many closely related species^31^. They are so named because some species utilize silk in constructing webbing on leaves or pads for oviposition and also for dispersal via ballooning much in the manner of some spiders. Spider mites have two reproductive strategies (bisexual and parthenogenetic). Many species of them have a wide host range, whereas others are highly host-specific. For example, *Tetranychus urticae*, *Tetranychus truncatus*, *Tetranychus kanzawai* and *Panonychus citri* are polyphagous and are serious pests of agricultural and horticultural crops. However, these genera also include oligophagous species, such as *Tetranychus bambusae* and *Oligonychus orthius* which inhabit only Poaceae plants. Previous studies have revealed that *Wolbachia* is widespread in spider mites. For example, *Wolbachia* has been detected in the genus *Tetranychus*^32^,^33^, *Oligonychus*^33^, *Panonychus*^33^, *Schizotetranychus*^33^, *Bryobia*^34^ and *Amphitetranychus*^35^, Furthermore, it was found associated with CI phenotypes in several species^8^,^26^,^27^,^32^,^33^,^36^ *Cardinium* was present in 15 species of family Tetranychidae, and induced CI in *Tetranychus piercei*^8^, *Tetranychus phaselus*^26^, *T. truncatus*^27^ and *Eotetranychus suginamensis*^37^. Unlike the widespread distribution of *Wolbachia* and *Cardinium*, *Rickettsia* and *Spiroplasma* were less common, they were only found in *T. urticae*^38^,^39^. As yet, comparative studies that focus on these endosymbionts in a group of spider mites species are very limited.

Here, we surveyed for the first time incidences of the four endosymbionts in economically important species of spider mites. Double infections of more than one endosymbiont were frequent within the same species, we then evaluated the levels of co-infection. We further clarified the phylogenetic relationships of these detected endosymbionts to infer their evolutionary histories. Our data provide insights into the evolution and distribution of endosymbionts in spider mites and may thus be regarded as a basis for future studies on spider mites and endosymbionts interactions.

## Results

### Incidences of tested endosymbionts in spider mites

**Figure 1:**
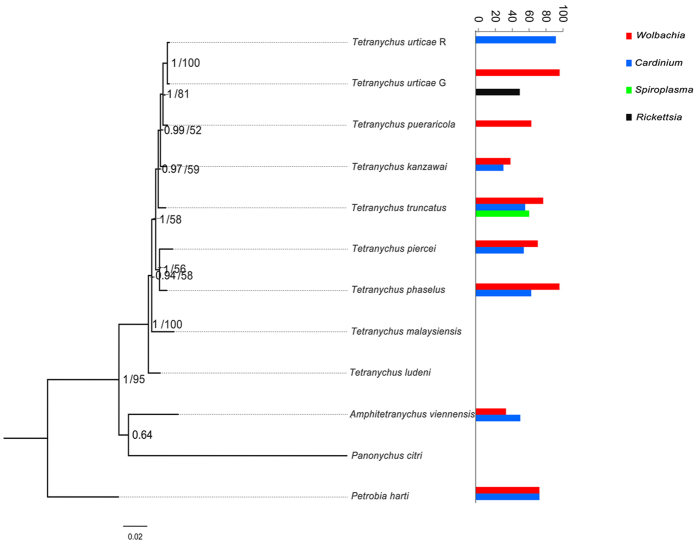
Phylogeny of spider mites based on *COI*, *18S* *rRNA*, *28S rRNA* gene sequences (left) and endosymbionts infection (right). Bayesian posterior (left numbers) and ML bootstrap values (right numbers, values >50% are indicated) are given in the tree. Prevalence of each endosymbiont is given.

Of the 12 spider mite species examined, *Wolbachia* was found to infect 8 species with prevalence ranging from 16.7 to 100%. *Cardinium* was found to infect 7 species and their infection frequencies ranging from 4.3 to 100% (Table 1). Among them, *Cardinium* infections in *T. kanzawai* and *Amphitetranychus viennensis* are new reports. *Wolbachia* infections were more frequent in *T. truncatus* than in *A. viennensis* (80.4% vs 36.5%, *P* < 0.05), while *Cardinium* infections showed no difference between them (58.9% vs 53.1%, *P* = 0.18). Other endosymbionts infections showed some host species-specificity, as *Spiroplasma* was found in *T. truncatus* and *Rickettsia* was detected in *T. urticae* G (Table 1, Fig. 1).

### Correlated infections with multiple endosymbionts

Of note, co-infections of unrelated endosymbionts were observed in several species. For instance, *Wolbachia* and *Cardinium* usually co-infect *T. truncatus*, *T. kanzawai*, *T. phaselus*, *T. piercei*, *A. viennensis* and *Petrobia harti*. Similarly, *T. urticae* G was infected by *Wolbachia* and *Rickettsia*, and *T. truncatus* showed triple infections (Table 1, Fig. 1). Furthermore, Spearman correlation analyses of the presence/absence of each endosymbiont within spider mite individuals against the presence/absence of other endosymbionts revealed that infections with *Wolbachia* and *Cardinium* were significantly correlated to each other (r = 0.4344, *P* < 0.01). Similarly, infections with *Wolbachia* and *Spiroplasma* were significantly correlated to each other (r = 0.4737, *P* < 0.01) in *T. truncatus*.

### Phylogeny of spider mites hosts and detected endosymbionts

Bayesian and maximum likelihood phylogenies of spider mites based on *COI*, *18SrRNA* and *28SrRNA* were identical. Species of the genus *Tetranychus* appeared to be monophyletic with strong support of posterior probabilities (>0.9) and moderate support of maximum likelihood bootstrap values (>50), other branches were not be well resolved (Fig. 1).

**Figure 2:**
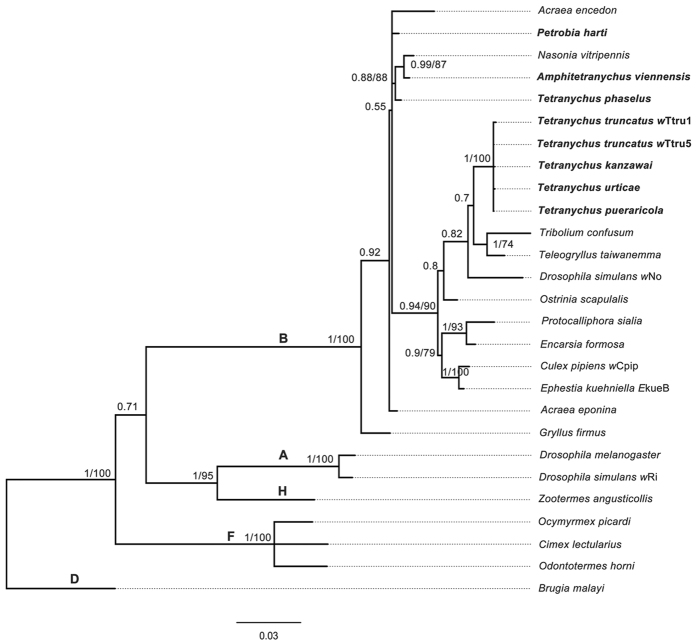
Bayesian inference phylogeny of *Wolbachia* based on the concatenated MLST data. The topology resulting from the Maximum Likelihood method was similar. The *Wolbachia* strains obtained from in this study are indicated in bold letters. Strains are characterized by the names of their host species. Bayesian posterior (left numbers) and ML bootstrap values (right numbers) are given (only values >50% are indicated).

Analyses of the *wsp* gene sequences in the seven positive species revealed nine *Wolbachia* strains, which were designated as *w*Phar, *w*Avie, *w*Tpha, *w*Turt, *w*Tpue, *w*Tkan, *w*Tpie, *w*Ttru1 and *w*Ttru5 (Table 1). These strains, except for *w*Tpie, were characterized by MLST. *Wolbachia* phylogenies based on MLST genes were largely identical for both Bayesian and maximum likelihood analyses, and most splits were highly supported. All of the *Wolbachia* strains from spider mites were assigned to supergroup B. *Wolbachia* strains from *T. truncatus*, *T. kanzawai*, *T. urticae* and *T. pueraricola* showed little divergence, and formed a monophyletic group (Fig. 2). While the *Wolbachia* strains obtained from *T. phaselus*, *A. viennensis* and *P. harti* respectively exhibited a distinct node in the phylogenies (Fig. 2).

**Figure 3:**
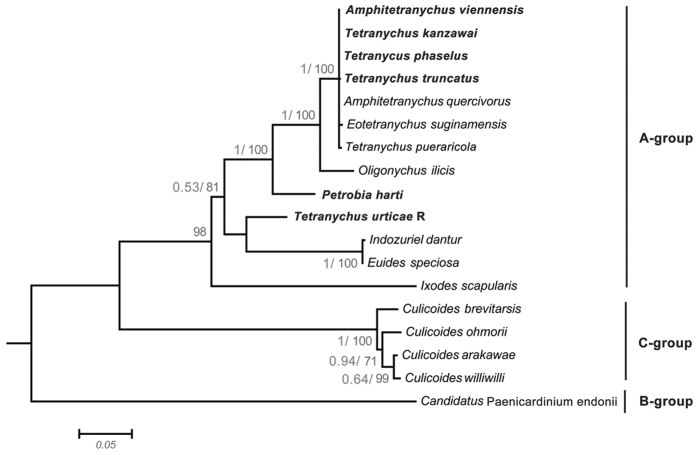
Phylogenetic analyses of *Cardinium* based on the *gyrB* gene sequences from this study (highlighted) and others downloaded from GenBank. *Cardinium* group names are used in accordance to the reference^25^. Bayesian posterior (left numbers) and ML bootstrap values (right numbers, values >50% are indicated) are given in the trees.

Based on the *16S rRNA* sequences, a total of seven *Cardinium* strains (*c*Tpie, *c*Phar, *c*Avie, *c*Tpha, *c*Turt, *c*Tkan and *c*Ttru) were found. Owing to the *16S rRNA*’s low discriminating ability, we performed phylogenetic analyses using the *gyrB* sequences. The *gyrB* gene of *c*Tpie was not successfully sequenced, which was therefore not represented in the *Cardinium* phylogeny. The remaining strains all belonged to group A-clade, and strains derived from *A. viennensis*, *T. kanzawai, T. phaselus* and *T. truncatus* plus with *Cardinium* from other spider mites species formed a monophyletic group (Fig. 3).

**Figure 4:**
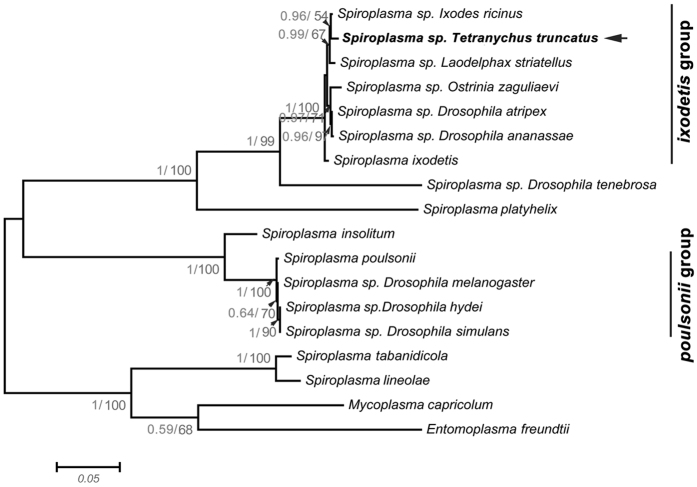
Phylogenetic analyses of *Spiroplama* based on the *rpoB* gene sequences. *Spiroplama* infecting *T. truncatus* is indicated in bold letters. Bayesian posterior (left numbers) and ML bootstrap values (right numbers, values >50% are indicated) are given in the trees.

**Figure 5:**
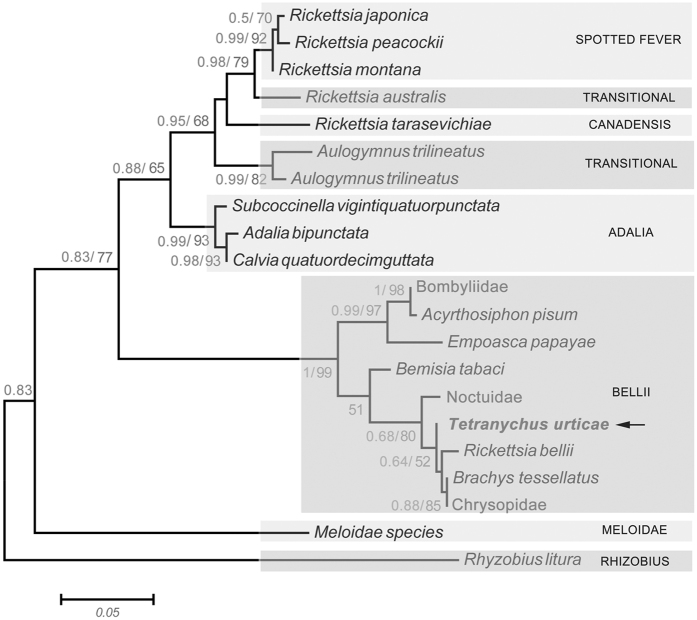
ML inference of *Rickettsia* strains from *T. urticae* and other arthropod hosts based on the *gltA* gene sequences. *Rickettsia* strain obtained from this study is indicated in bold letters. *Rickettsia* group names are used in accordance to the reference^39^. Bayesian posterior (left numbers) and ML bootstrap values (right numbers, values >50% are indicated) are given in the trees.

Sequencing of the *Spiroplasma*’s *16S rRNA*, *rpoB* gene and the *Rickettsia*’s *gltA* gene identified one *Spiroplasma* strain from *T. truncatus* and one *Rickettsia* strain from *T. urticae* G, respectively (Table 1, Table S2). Phylogenetic tree based on *rpoB* gene demonstrated that the *Spiroplasma* of *T. truncatus* fell into the ixodetis group, which includes *Spiroplasma ixodetis* and *Spiroplasma* infecting tick, planthopper, moth and flies (Fig. 4). The *Rickettsia* detected from *T. urticae* G fell within the bellii group (Fig. 5).

### Correlation between *Wolbachia*, *Cardinium* and hosts genetic distances

The pattern of association and genetic divergence between *Wolbachia*, *Cardinium* and hosts were examined. Pairwise genetic distances of hosts and associated *Wolbachia* were significantly correlated (r = 0.4828, *P* = 0.005). While there was no significant correlation between *Cardinium* and hosts genetic distances (r = 0.1939, *P* = 0.29).

## Discussion

Studies of endosymbiont’ incidences in a wide variety of arthropods suggest that *Wolbachia* is the most common bacterial symbiont^20^. Acari is assumed to be a hotspot for *Wolbachia* infections^25^,^29^. The finding of about 67% *Wolbachia*-positive species in our study is in line with estimations of a general *Wolbachia* prevalence among arthropods (40–60%). *Cardinium* infections were identified in 7 out 12 species (58.3%), suggesting that spider mites are prone to be infected with *Cardinium*, which is consistent with previous estimates^23^,^24^,^40^. Whereas *Spiroplasma* and *Rickettsia* showed host species-specificity, they were only detected in *T. truncatus* and *T. urticae*, respectively. Ecological traits of host can affect the infection dynamics of endosymbionts in them^41^,^42^. Although the infection frequencies of these endosymbionts varied among geographical populations, our survey data did not detect a clear correlation between their distribution and host ecological traits (Table 1). It is worth noting that three lab reared lines of *T. urticae* G were completely infected with *Wolbachia*, indicating fixation of infection has been reached in rearing. In addition, three species *T. malaysiensis*, *T. ludeni*, *P. citri* did not carry any of the tested endosymbionts, raising the possibility that there have been repeated losses of infection during post-speciation from an infected ancestor or due to limited samples. *Wolbachia* infections were more frequent in *T. truncatus* than in *A. viennensis*. The two species are phylogenetically divergent and have different habitats, thus it remains possible that host phylogeny combined with host ecology shapes the distribution of endosymbionts in spider mites.

Manipulating reproduction and providing fitness advantages in their hosts are thought to be two important determinants of endosymbionts infection frequencies^43^. The high infection frequencies of *Wolbachia* and *Cardinium* in spider mites may be due to their reproductive manipulations or fitness advantages. Nine *Wolbachia* strains were detected from the positive specimens, and several of these strains have previously been studied in detail. For example, there is evidence that *Wolbachia* induces CI in several spider mites, including *T. urticae*^32^,^33^,^43^, *T. phaselus*^26^, *T. truncatus*^27^, *T. piercei*^8^ and *A. viennensis*^35^. Regarding *Cardinium*, it was found to induce CI in *T. piercei*^8^, *T. phaselus*^26^ and *T. truncatus*^27^. Furthermore, Weinert *et al*.^40^ have speculated that high *Cardinium* incidences in spider mites might reflect evolutionary changes in arthropod immunity, as spider mites lack components of the immune deficiency (IMD) pathway, and IMD is activated by diaminopimelic acid-type (DAP-type) peptidoglycan, which is produced by *Cardinium*^44^.

Spider mites showed co-infections with more than one endosymbiont. Statistical analyses revealed that infections with *Wolbachia* and *Cardinium*, *Wolbachia* and *Spiroplasma* were significantly correlated to each other within the same individuals. There are a number of possible mechanisms that can facilitate such endosymbiont co-infections^45^. For example, the co-infecting endosymbionts may additively or synergistically confer fitness advantages on their host. Another mechanism is that when one of the co-infecting endosymbionts causes a reproductive manipulation, the manipulation may facilitate not only its own prevalence but also spread of another co-infecting endosymbiont via a hitchhiking effect^46^. As mentioned previously, both *Wolbachia* and *Cardinium* can induce CI in doubly-infected spider mites *T. piercei*^8^, *T. phaselus*^26^, *T. truncatus*^27^ and *A. viennensis*^35^, which would increase the prevalence of both endosymbionts. Also, the CI phenotypes induced by *Wolbachia* in *T. truncatus* and *T. urticae* G would theoretically facilitate the spread of co-infecting *Spiroplasma* and *Rickettsia*, respectively.

*Spiroplasma* and *Rickettsia* act as reproductive mediators in some arthropods^11^,^12^,^13^,^14^,^15^,^16^,^17^. They are much less common in spider mites than *Wolbachia* and *Cardinium*. Here, *Spiroplasma* and *Rickettsia* were identified only in *T. truncatus* and *T. urticae* G, respectively. Phylogenetic analyses confirmed that the two endosymbionts have horizontally transferred among different hosts, and there is experimental evidence for horizontal transmission of these symbionts via host plants or host invertebrates^47^,^48^, thus *Spiroplasma* and *Rickettsia* occurrences in spider mites may be the result of an individual horizontal transmission event, respectively. Furthermore, *Spiroplasma* of *T. truncatus* fell into the ixodetis group, which includes *Spiroplasma ixodetis* and *Spiroplasma* infecting tick, planthopper, moth and flies. Unlike the male-killing *Spiroplasmas* infecting the small brown planthopper, *Laodelphax striatellus*^14^, the *Spiroplasma* strain found in *T. truncatus* is not a male killer because it was found in both males and females. While it seemed increase its host development speed (unpublished data), and we are currently testing the underlying reasons. *Rickettsia* in *T. urticae* clustered within the bellii group, in which there are *Rickettsia* strains found in sap sucking arthropods and predatory insect hosts. Previously, Hoy and Jeyaprakash^49^ also found that four North American populations of *T. urticae* were infected with *Rickettsia*, as well as *Wolbachia* and *Caulobacter*. However, what role the *Rickettsia* play in the biology of spider mites is unknown.

Theory suggested that maternally inherited symbiontes’ strict mutualistic associations with their hosts will result in co-cladogenesis phylogenetic patterns^50^,^51^. Multiple strains of *Wolbachia* and *Cardinium* were detected in this study, and their infection histories could be inferred. Mapping the *Wolbachia* phylogeny to spider mites’ phylogeny revealed some degree of congruence, as similar strains are found in closely related hosts. Meanwhile, pairwise genetic distances of hosts and associated *Wolbachia* were significantly correlated. Two scenarios might explain this finding. First, the common ancestor of spider mite hosts could have originally harbored *Wolbachia* and that the host and *Wolbachia* have co-speciated. Second, horizontal transmission can explain the sharing of *Wolbachia* strains among different hosts. By contrast, there was no significant association between the phylogeny of *Cardinium* and its host, and the Mantel test confirmed this result. Whilst we cannot completely exclude the possibility that there has been repeated loss of infection during post-speciation from an infected ancestor, this difference between the two phylogeneies indicated that *Cardinium* was not solely acquired vertically and points to the likelihood of horizontal transmission. In addition, the phylogeny based on a single gene will fail to reflect the accurate phylogeny of *Cardinium*, thus more rapidly evolving and phylogenetically informative *Cardinium* genes are required in further study.

In summary, four endosymbionts were identified in the tested specimens, and their distributions were found to be shaped by both host phylogeny and host ecology. The levels of co-infections within the same individuals were significantly higher than would be expected by chance. Comparison between the phylogenies of hosts and associated endosymbionts allowed us explore the evolutionary histories of these endosymbionts’ infections. Together with these endosymbionts’ reproductive effects on spider mites, these findings are helpful for understanding the interaction between endosymbionts and spider mites.

## Methods

All experimental protocols were approved by Chinese Academy of Sciences and Nanjing Agricultural University. Methods were carried out in accordance with relevant guidelines and regulations.

### Spider mites samples

This study was based on specimens from 28 populations of spider mites representing 12 species that were collected from field or lab reared lines (Table 1). All samples were stored in 100% ethanol and frozen at −20  C until DNA extraction.

### PCR screening and sequencing

Spider mite DNA was extracted as previously described^52^. The DNA quality was tested by amplifying a fragment of the cytochrome oxidase, subunit I (*COI*) gene of spider mites^53^. Then, the presence of *Wolbachia*, *Cardinium*, *Spiroplasma* and *Rickettsia* was assessed by PCR amplification using specific primers and annealing temperatures listed in Table S1. PCRs were carried out on a Veriti machine (ABI Biosystems, USA) in 25 μl volume containing 12.5 μl 2 × Taq Master Mix (Vazyme Biotech, China), 0.5 μl primer (20 μM each), 1 μl of DNA extract. Positive and negative controls were included in PCR reactions. PCR products (5 μl) were visualized on a 1.5% agarose gel stained with ethidium bromide^54^. The positive products were purified using AxyPrep DNA Gel Extraction kit (AxyGEN, USA) and then directly sequenced (Majorbio Company, Shanghai, China). For *Wolbachia*, single-infection status was confirmed during *wsp* sequencing. Then, the MLST gene sequences of single infected *Wolbachia* from different individuals were amplified using standard primers and PCR protocols (http://www.pubmlst.org/wolbachia/). The *COI*, *18SrRNA* and *28SrRNA* sequences of spider mites were amplified and sequenced to construct host phylogeny. The obtained sequences have been deposited in GenBank (Table S2).

### Phylogenetic analysis

All sequences were aligned and manually corrected using BioEdit^55^. Prior to phylogenetic analysis, the best-fitting nucleotide models were determined by jModeltest version 2^56^. *Wolbachia* phylogeny was determined by reconstructing Bayesian Inference (BI) and Maximum-Likelihood (ML) trees of the concatenated data set of MLST genes from this study and PubMLST database (http://www.pubmlst.org/wolbachia/). Bayesian analyses were performed in MrBayes 3.1^57^ under the GTR + I + G model. Four Markov chains were run for 15,000,000 generations with sampling every 100 generations, and the first 37,500 generations were discarded. Convergence of runs was assumed when split frequencies reached <0.01. ML analysis was also conducted for the concatenated data set of MLST genes in MEGA 5.0^58^ under the GTR + I + G model, bootstrap pseudoreplicates were calculated 1,000 times.

The phylogenetic analyses of *Cardinium*, *Spiroplasma* and *Rickettsia* were performed using Bayesian Inference (BI) and Maximum-Likelihood (ML) estimation for the sequences of *gyrB* gene, *rpoB* gene and *gltA* gene, respectively. The evolutionary models used were as follows: *gyrB*- GTR + I + G, *rpoB*-GTR + I + G, and *gltA*-HKY+G. For each Bayesian analysis, four Markov chains were run 1,000,000 generations with sampling every 100 generations, and the first 25% of samples were discarded as burn-in. Convergence of runs was assumed when split frequencies reached <0.01. ML analyses were conducted in MEGA 5.0^58^, bootstrap pseudoreplicates were calculated 1,000 times.

In addition, Bayesian and ML phylogenetic trees of spider mite were constructed using a supermatrix that consisting of the *COI*, *18SrRNA* and *28SrRNA* sequences. Analyses were performed in MrBayes 3.1 and MEGA 5.0 under the GTR+G+I model, respectively.

### Statistical analysis

Endosymbiont infection patterns were first compared on the level of genera and species of spider mites. Two extensively sampled species, *T. truncatus* and *A. viennensis* were selected to test the differences of *Wolbachia* and *Cardinium* infections. Because co-infection was common in spider mites, co-infection levels were evaluated with a Spearman correlation analysis of the presence/absence of each endosymbiont within spider mite individuals against the presence/absence of other endosymbionts. The analysis was performed with Stata 11.0^59^. In addition, we tested for correlation between host genetic distance and the corresponding *Wolbachia*, *Cardinium* strains genetic divergence to infer their infection patterns. Correlation analyses were performed using a Mantel test in Arlequin 3.1^60^ with 1000 permutations. Genetic distance matrices of the concatenated MLST dataset for *Wolbachia*, *gyrB* genes for *Cardinium* and the concatenated nuclear and mitochondrial loci for spider mites were calculated with the Kimura 2-parameter model in MEGA 5.0.

## Additional Information

**How to cite this article**: Zhang, Y.-K. *et al*. Screening of spider mites (Acari: Tetranychidae) for reproductive endosymbionts reveals links between co-infection and evolutionary history. *Sci. Rep.* **6**, 27900; doi: 10.1038/srep27900 (2016).

## Supplementary Material

Supplementary table S1 S2

## Figures and Tables

**Table 1:** Prevalence of investigated endosymbionts in different lines of spider mites.

Population code	Species	Host plant	Location	Collection date	Collection source	Endosymbionts infection^*^			
						*Wolbachia*% infected	*Cardinium*% infected	*Spiroplasma*% infected	*Rickettsia*% infected
1	*Tetranychus truncatus*	Cotton	Harbin, Heilongjiang	Aug-2011	Field collected	100 (24/24) *w*Ttru5	100 (24/24) *c*Ttru	83.3 (20/24) *Spiroplasma sp*.	–
2		Eggplant	Changchun, Jilin	Jul-2014	Field collected	–	100 (12/12) *c*Ttru	–	–
3		Japan Caryatia	Yanji, Jilin	Aug-2011	Field collected	100 (24/24) *w*Ttru1	12.5 (3/24) *c*Ttru	37.5 (9/24) *Spiroplasma sp*	–
4		Mung bean	Shenyang, Liaoning	Aug-2011	Field collected	69.6 (16/23) *w*Ttru5	4.3 (1/23) *c*Ttru	82.6 (19/23) *Spiroplasma sp*	–
5		Bean	Hohhot, Inner Mongolia	Aug-2014	Field collected	100 (12/12) *w*Ttru1	100 (12/12) *c*Ttru	100 (12/12) *Spiroplasma sp*	–
6		Eggplant	Jiuquan, Gansu	Sep-2012	Field collected	75 (9/12) *w*Ttru5	–	–	–
7		Snake gourd	Cangzhou, Hebei	Aug-2014	Field collected	100 (24/24) *w*Ttru1	100 (24/24) *c*Ttru	100 (24/24) *Spiroplasma sp*	–
8		Eggplant	Changzhi, Shanxi	Aug-2014	Field collected	100 (20/20) *w*Ttru1	100 (20/20) *c*Ttru	100 (20/20) *Spiroplasma sp*	–
9		Corn	Chuzhou, Anhui	Sep-2010	Field collected	16.7 (2/12) *w*Ttru1	–	–	–
10	*Tetranychus kanzawai*	Chinese rose	Qingdao, Shandong	Jun-2011	Field collected	41.7 (5/12) *w*Tkan	33.3 (4/12) *c*Tkan	–	–
12	*Tetranychus urticae* (Green form)	Apple	Taian, Shandong	Aug-2014	Lab reared	100 (12/12) *w*Turt	–	–	75 (9/12) *Rickettsia sp*
13		Bean	Hohhot, Inner Mongolia	Aug-2014	Lab reared	100 (12/12) *w*Turt	–	–	100 (10/12) *Rickettsia sp*
14		Apple	Missouri, USA	Jul-2012	Lab reared	100 (12/12) *w*Turt	–	–	–
15	*Tetranychus urticae* (Red form)	Willow	Kunming, Yunnan	Jul-2012	Lab reared	*−*	95.8 (23/24) *c*Turt	–	–
16		Bean	Ibaraki, Japan		Lab reared	–	–	–	–
17	*Tetranychus pueraricola*	Purple yam	Yongfu, Guangxi	Aug-2014	Field collected	66.7 (8/12) *w*Tpue	–	–	–
18	*Tetranychus phaselus*	Bean	Quanzhou, Fujian	Jun-2014	Field collected	100 (12/12) *w*Tpha	66.7 (8/12) *c*Tpha	–	–
19	*Tetranychus malaysiensis*		Lingshui, Hainan	Jun-2014	Field collected	–	–	–	–
20	*Tetranychus piercei*	Kidney bean	Mayang, Hunan	Jul-2014	Field collected	75 (9/12) *w*Tpie	58.3 (7/12) *c*Tpie	–	–
21	*Tetranychus ludeni*	Melon	Shantou, Guangdong	Jul-2014	Field collected	–	–	–	–
22	*Amphetetranychus viennensis*	Plum	Daqing, Heilongjiang	Aug-2012	Field collected	–	–	–	–
23		Cherry	Yanji, Jilin	Aug-2012	Field collected	58.3 (14/24) *w*Avie	66.7 (16/24) *c*Avie	–	–
24		Purple-leaf plum	Zhengzhou, Henan	Jul-2012	Field collected	16.7 (4/24) *w*Avie	–	–	–
25		Purple-leaf plum	Sanmenxia, Henan	Jun-2012	Field collected	–	83,3 (20/24) *c*Avie	–	–
26		Peach	Nanjing, Jiangsu	Aug-2014	Field collected	70.8 (17/24) *w*Avie	62.5 (15/24) *c*Avie	–	–
27	*Petrobia harti*	Clover	Nanjing, Jiangsu	Oct-2010	Field collected	75 (9/12) *w*Phar	75 (9/12) *c*Phar	–	–
28	*Panonychus citri*	Citrus	Suzhou, Jiangsu	Sep-2010	Field collected	–	–	–	–

^a^The infection rate of each endosymbiont was presented. Data in the bracket indicates the number of infected individuals and the number of test individuals, respectively. – indicates endosymbionts were not detected.
