# Supplementary material for: Screening of spider mites (Acari: Tetranychidae) for reproductive endosymbionts reveals links between co-infection and evolutionary history
Source: Sci Rep. 2016 Jun 13;6:27900. doi: 10.1038/srep27900 (PMC4904281; doi:10.1038/srep27900)
Supplement: Supplementary table S1 S2 [file srep27900-s1.pdf]

Screening of spider mites (Acari: Tetranychidae) for reproductive endosymbionts reveals links between co-infection and evolutionary history

Yan-Kai Zhang[1, 2], Ya-Ting Chen[2], Kun Yang[2], Ge-Xia Qiao[1*] & Xiao-Yue Hong[2*]

[1] Institute of Zoology, Chinese Academy of Sciences, Beijing, 100101, China.

[2] Department of Entomology, Nanjing Agricultural University, Nanjing, Jiangsu 210095, China

[*]Corresponding author.

Ge-Xia Qiao

Institute of Zoology

Chinese Academy of Sciences

Beijing, 100101

China

Tel / Fax: 0086-10-64807133 / 0086-10-64807099

E-mail address: qiaogx@ioz.ac.cn

Xiao-Yue Hong

Department of Entomology

Nanjing Agricultural University

Nanjing, Jiangsu 210095

CHINA

Tel & Fax: 0086-25-84395339

E-mail address: xyhong@njau.edu.cn

Table S1. Primers used in this study

Organism	Gene	Product	Primer (5'-3')	Annealing temperature	Reference
Wolbachia pipients	*wsp*	Outer surface protein	wspF1-GTCCAATARSTGATGARGAAAC<br>wspR1-CYGCACCAAYAGYRCTRTAAA	55℃	61
	gatB	Glutamyl-tRNA(Gln) amidotransferase, subunit B	gatBF-GAKTTAAAYCGYGCAGGBGTT<br>gatBR-TGGYAAYTCRGGYAAAGATGA	54℃	61
	coxA	Cytochromecoxidase, subunit I	coxAF-TTGGRGCRATYAACTTTATAG<br>coxAR-CTAAAGACTTTKACRCCAGT	55℃	61
	hcpA	Conserved hypothetical protein	hcpAF-GAAATARCAGTTGCTGCAAA<br>hcpAR-GAAAGTYRAGCAAGYTCTG	53℃	61
	ftsZ	Cell division protein	ftsZF-ATYATGGARCATATAAARGATAG<br>ftsZR-TCRAGYAATGGATTRGATAT	54℃	61
	fbpA	Fructose-bisphosphate aldolase	fbpAF-GCTGCTCCRCTTGGYWTGAT<br>fbpAR-CCRCCAGARAAAAYYACTATTC	59℃	61
Cardinium hertigii	*16S rRNA*	16S ribosomal RNA	Ch-F-TACTGTAAGAATAAGCACCGGC<br>Ch-R-GTGGATCACTTAACGCTTTCG	57℃	24
	gyrB	Gyrase B subunit	gyrB1F-CAAAGAYACCTATAARAT TTCTG<br>gyrB1R-GTAACGTTGTACARAKACRGCAT	52℃	25
Spiroplasma sp.	*16S rRNA*	16S ribosomal RNA	SpitsJ04-GCCAGAAGTCAGTGTCCTAACCG<br>SpitsN55-ATTCCAAGGCATCCACCATACG	56℃	47
	rpoB	RNA Polymerase B	rpoBF3-GGNTTTATTGAAACACCATAYCGTC<br>rpoBR2-GCATGTAATTTATCATCAACCATGTGTG	55℃	62
Rickettsia sp.	*gltA*	Citrate synthase	RICS741F-CATCCGGAGCTAATGGTTTTGC<br>RCIT1197R-CATTTCTTTCCATTGTGCCATC	56℃	63

Spider mite	*COI*	Cytochromecoxidase, subunit I	COIF-TGATTTTTTGGTCACCCAGAAG<br>COIR-TACAGCTCCTATAGATAAAAC	52℃	53
	18S rRNA	18S ribosomal RNA	18S1F-ACCGCGAATGGCTCATTAAATCAGTT<br>18S10R-ACAAAGGGCAGGGACGTAATCAA	56℃	64
	28S rRNA	28S ribosomal RNA	28v5'-AAGGTAGCCAAATGCCTCATC<br>28jj3'-AGTAGGGTAAAACTAACCT	52℃	64

Table S2. The GenBank accession numbers for sequences obtained in this study

	Spider mite			*Wolbachia pipients*						*Cardinium hertigii*		*Spiroplasma sp.*		*Rickettsia sp.*
Spider mite species	*COI*	*18S rRNA*	*28S rRNA*	*wsp*	*gatB*	*coxA*	*hcpA*	*ftsZ*	*fbpA*	*16S rRNA*	*gyrB*	*16S rRNA*	*rpoB*	*gltA*
Tetranychus truncatus	JX094361	KU323529	KU323541	JX094379	JX094425	JX094412	JX094409	JX094402	JX094395	KP828054	KU323554	KP828067	KU323561	
				JX094383	JX094426	JX094421	JX094409	JX094402	JX094395	KP828054	KU323554	KP828067	KU323561	
Tetranychus kanzawai	KP828065	KU323532	KU323544	JX094391	JX094426	JX094419	JX094409	JX094402	JX094395	KP828053	KU323552			
Tetranychus urticae Green form	KP828062	KU323534	KU323546	JX094388	JX094426	JX094415	JX094409	JX094402	JX094395					KP828066
Tetranychus urticae Red form	KP828063	KU323530	KU323542							KP828050	KU323550			
Tetranychus pueraricola	KP828064	KU323536	KU323548	KP828068	JX094426	JX094421	JX094409	JX094402	JX094395					
Tetranychus phaselus	KP828060	KU323527	KU323539	KP828069	JX094426	JX094416	JX094406	JX094403	JX094397	KP828051	KU323553			
Tetranychus malaysiensis	KP828058	KU323531	KU323543											
Tetranychus piercei	KP828061	KU323526	KU323538	KU323560	-	-	-	-	-	KU323562	-			
Tetranychus ludeni	KP828059	KU323528	KU323540											
Amphetetranychus viennensis	JX094368	KU323535	KU323547	JX094392	JX094423	JX094417	JX094411	JX094403	JX094398	KP828052	KU323551			
Petrobia harti	KP828056	KU323525	KU323537	KP828070	KU323555	KU323556	KU323557	KU323558	KU323559	KP828055	KU323549			
Panonychus citri	KP828057	KU323533	KU323545											
